# Oncological Outcomes in Rats Given Nephrocarcinogenic Exposure to Dietary Ochratoxin A, Followed by the Tumour Promoter Sodium Barbital for Life: A Pilot Study

**DOI:** 10.3390/toxins2040552

**Published:** 2010-03-31

**Authors:** Peter G. Mantle, Miloslav Dobrota, Cheryl E. Gillett, Edward W. Odell, Sarah E. Pinder

**Affiliations:** 1 Centre for Environmental Policy, Imperial College London, London, UK; 2 Faculty of Health and Medical Sciences, University of Surrey, Guildford, Surrey, UK; Email: miloslav.d@googlemail.com; 3 Hedley Atkins Breast Pathology Laboratory, Guy’s Hospital, London, UK; Email: cheryl.gillett@kcl.ac.uk; 4 Department of Oral Pathology, King’s College London, London, UK; Email: edward.odell@kcl.ac.uk; 5 Department of Academic Oncology, Guy’s Hospital, London. UK; Email: sarah.pinder@kcl.ac.uk

**Keywords:** DNA ploidy, latency, mononuclear leukaemia, renal tumour, mammary tumour, angiosarcoma

## Abstract

The potent experimental renal carcinogenesis of ochratoxin A (OTA) in male rats makes the dietary contaminant a potential factor in human oncology. We explored whether the tumour promoter sodium barbitate could shorten the otherwise long latency between exposure to toxin and tumourigenesis. Young rats, of a hybrid in which mononuclear leukaemia was rare, were given feed contaminated (5 ppm) with OTA for 36 weeks to initiate renal tumourigenesis. Some individuals were thereafter given sodium barbitate (500 ppm in drinking water) for life. Pathological outcomes were studied at or near the end of natural life. Renal tumours in males given barbitate became evident after latency of one year, but only slightly before those without barbitate. In contrast, female mammary tumourigenesis was advanced by at least 6 months synchronously in all rats given the OTA-barbitate regimen compared to tumourigenesis in controls. Diagnosis of malignant mammary angiosarcoma in a female given the OTA-barbitate regimen is a new finding in the rat. The long latency of OTA-induced renal tumourigenesis was not notably susceptible to accelerated promotion by barbitate, contrasting with an apparently marked effect of barbitate on development of mammary tumours.

## 1. Introduction

The renal carcinogenicity of ochratoxin A (OTA) has been well demonstrated in several 2-year studies [1,2,3] with particular reference to male rats of the Fischer, Lewis and Dark Agouti strains. OTA appears to be a complete carcinogen, requiring no other particular xenobiotic as a tumour promoter, but rat renal tumours are usually only discovered in the last quarter of natural life even under continuous lifetime regimens of oral gavage or dietary contamination. The opportunity to explore tumour promotion for this toxin has been provided by recent findings that exposure to OTA is necessary for no more than 9–10 months, from about 8–10 weeks of age, in order to have individuals programmed to inevitable tumourigenesis [4]. Tumours still become apparent in the last quarter of natural life, but this involves a latency of about a year during which the toxin has long been absent.

If, by whatever mechanism, permanent genetic change gradually occurs in the kidney due to OTA while it is circulating in blood, response to subsequent exposure to potential promoters could be tested by measuring temporal advance in tumourigenesis during the second year of life. Although OTA has not been proved to have caused any human disease there is concern that humans could be at risk. However, at present there is no evidence for etiological similarity between experimental renal tumours in male rats and those (mostly idiopathic) in humans; to understand OTA-induced renal tumour promotion can contribute to that objective. Our present study has been made on a pilot scale to explore any accelerated tumourigenesis in rats treated first with an OTA regimen expected to be sufficient to cause renal tumours at least in males. We chose to use a type containing only part of the F344 (Fischer) rat genome in order hopefully to minimise the confounding factor of spontaneous leukaemia during ageing, which can cause premature death of up to half of the animals. However, it was thought desirable to conserve some of the Fischer genome to assist comparison with data from NTP protocols.

Evidence for tumourigenic potential of OTA in female rats is somewhat equivocal; the NTP study [1] showed increased incidence of the typical naturally-occurring mammary fibroadenomas only at the highest OTA dose. Thus the present study has included females for the first time in a lifetime study with dietary OTA, not only to compare plasma OTA pharmacodynamics but also to observe any mammary tumourigenesis. 

The potential role of barbiturates as tumour promoters is well recognised [5,6,7], particularly targeting the liver where marked response was evident within a week of exposure to dietary phenobarbital [7]. Extension of influence to renal tumourigenesis was demonstrated concerning promotion by sodium barbital (500 ppb in drinking water) of multiple cortical adenomas and distant metastases, initiated by a single injection of nickel (ll) acetate [8]. Effects were evident at necropsy amongst decedents where ~40% of treated male F344 rats had died by 85 weeks of age. More recently, sodium barbital has been shown to induce early development of the renal lesions that spontaneously arise in *Tsc2* mutant (Eker) male rats [9]. Eker rats have also been of interest as potential short-term models for study of ochratoxin-initiated renal tumours [10]. Thus, sodium barbitate was chosen as the exogenous tumour promoter for the present study.

## 2. Materials and Methods

### 2.1. Experimental animals

**Figure 1:**
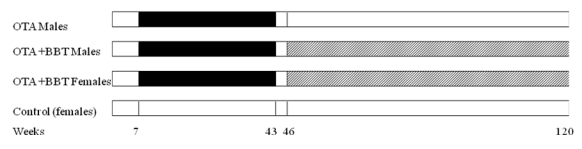
Diagrammatic scheme showing exposures of groups of rats to dietary OTA (5 ppm) [black] or sodium barbitate in drinking water (500 ppb) [shaded]. Blank regions indicate standard rat diet (14% protein). The 120 week mark on the x-axis represents the stage of general high mortality, although the oldest surviving animal lived for 150 weeks (Figure 2).

Two Sprague Dawley Female rats were crossed with a Fischer (F 344) male. The latter proved to have been a carrier of genetic susceptibility for developing the typical spontaneous mononuclear leukaemia during ageing. Litters were born within 4 days of each other. The outline of subsequent experimental regimens is given in Figure 1. When 7–8 weeks old, ten males and three females one litter were given a 5 ppm OTA diet (20 g consumed daily, delivering 100 µg OTA): for 36 weeks, caged in groups as previously described [3]. Standard feed throughout was SDS Services RM1 diet (14.4% protein). Three control females were maintained together through life on the standard diet. Weight was subsequently monitored through life.

After 22 weeks of the OTA treatment, and also just before completion at 36 weeks, all females and four males were maintained individually in metabolism cages overnight (18 hours, with feed and water *ad libitum*) for assessment of renal function, first by measurement of urine output. Urine was stored at –20 °C for subsequent urinalysis.

After 32 weeks of OTA treatment, blood samples were taken from a tail vein. Blood was sampled similarly on completion of OTA treatment and at intervals thereafter to follow the plasma half-life of OTA. 

Three weeks after OTA exposure ceased, five males were selected randomly from the group of ten and were given sodium barbitate (500 ppm w/v in drinking water *ad libitum*) for life. The other five males were returned to standard diet. The females given OTA continued on to the barbitate regimen. Animals were culled whenever health was seen to deviate from acceptable standards according to UK Home Office requirements. Post-mortem examination in each case focused primarily on overt reasons for cull, e.g., the splenomegaly of leukaemia, a large subcutaneous tumour, macroscopic features of a renal tumour, metastatic nodules on abdominal serosal surfaces, or abnormalities of testes or mammary tissues. 

### 2.2. Urinalysis

Automated urinalysis for creatinine, protein, calcium, sodium, potassium, urate, urea and phosphate was performed in the Chemical Pathology Laboratory at St. Mary’s Hospital, Paddington, London, using an Olympus AU640 instrument with methodology described in [11]. 

### 2.3. OTA in blood plasma

Quantitative measurement was made at the Central Science Laboratory, York by validated methodology as previously described [4]. 

### 2.4. Histopathology

Standard wax-embedded blocks were prepared from kidneys and all tumour tissues, and sections (3–4 µm) were stained with haematoxylin and eosin in the Breast Pathology laboratory, Guy’s Hospital, London.

### 2.5. DNA ploidy measurement

Ploidy analysis was performed as previously described [12]. H and E sections from blocks were examined and maximum areas for analysis marked to guide excision of corresponding regions from wax sections. Briefly, three to six 50 µm sections were cut, dewaxed in xylene, rehydrated and nuclei extracted using protease type XXIV (Sigma Chemical Co., Poole, UK) as described in [13]. Nuclear monolayers were prepared in a cytospin 4 (Thermo Shandon, UK) and stained by the Feulgen-Schiff method and analysed on a Fairfield image-based ploidy analyzer (Medical Solutions, UK) based on a Zeiss Axioplan II microscope (Zeiss, Germany). Monolayers with a minimum of 300 nuclei sampled were analysed using the automated scanning option.

Diagnostic criteria for ploidy status were as previously published for this system [12]. A sample was classified as diploid if only one G0/G1 (2c; conventional nomenclature in ploidy analysis for diploid, equivalent to 2n) peak was present, or if the number of nuclei in the G2 (4c; equivalent to tetraploid) peak did not exceed 10% of the total number of epithelial cells. A sample was defined as DNA tetraploid when the fraction of nuclei in the 4c region exceeded 10% of the total, without corresponding S phase. A lesion was defined as aneuploid if non-euploid peaks were present, or if the number of nuclei with DNA content greater than 5c exceeded 1%. A sample was classified as being indeterminate if less than 300 nuclei suitable for analysis were obtained, or if insufficient material remained for analysis. Specimens of known diploid and aneuploid DNA content acted as technique controls and produced the expected results. 

## 3. Results

**Figure 2:**
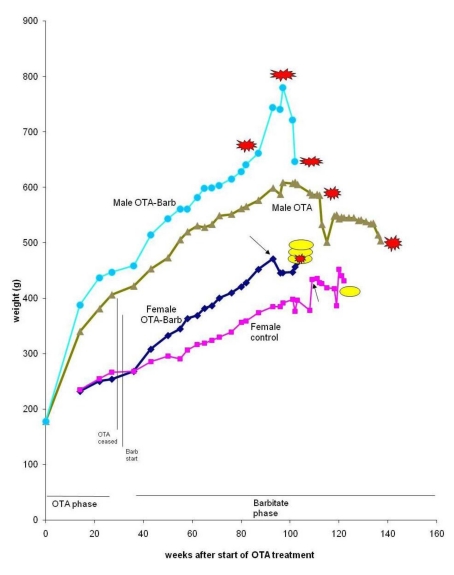
Mean weight of groups of male and female rats throughout life from commencement of the experiment. Parallel vertical lines show a 3-week untreated period separating the initial period of exposure to dietary OTA from subsequent treatment with sodium barbitate. Red stars represent discovery of renal tumours. Yellow oval shapes indicate euthanized rats with mammary tumours.

Generally, neither the 36 weeks of continuous ingestion of dietary OTA nor subsequent treatment with sodium barbitate caused any clinical abnormalities in rats of either gender. On the contrary, start of the barbitate treatment was associated with immediate increase in the rate of body weight accumulation, particularly in the females (Figure 2), evident long before attributable to tumour proliferation.

Occurrence of only a single case of leukaemia, which could probably not have been diagnosed at the 2-year terminal stage typical for NTP protocols, shows that the present rat hybrid may be useful in lifetime toxicological studies to conserve part of the Fischer genome while avoiding the problem of premature deaths; incidence can be up to 50% in the Fischer strain [14] due to mononuclear leukaemia [15]. 

### 3.1. OTA in blood

**Figure 3:**
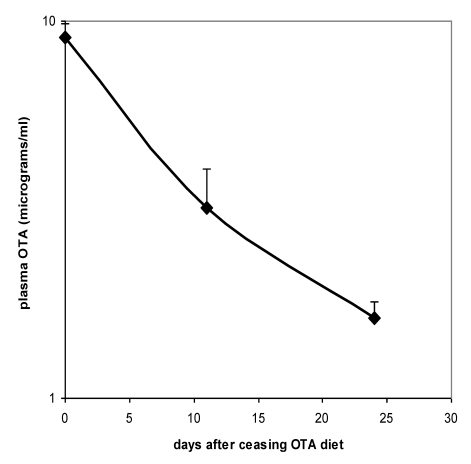
Plasma OTA half-life curve for Sprague-Dawley x Fischer hybrid female rats upon ceasing 36 weeks exposure to 5 ppm dietary OTA. Error bars show standard deviation.

Proof of the level of circulation of OTA in both males and females during the potentially tumourigenic first phase of the study was obtained towards its end at the 32 week stage. Close correlation between plasma OTA concentration in each of the 3 replicates analysed allowed confidence in the gender difference, in which males had a mean concentration of 6.03 µg/mL (SD 0.50) and females 11.17 µg/mL (SD 0.29). On account of body weight difference between genders and the slightly lower daily feed intake of females, it is calculated that males were ingesting less OTA than females in proportion to body weight (~150 µg/kg body weight *versus* ~200 µg/kg, respectively). Nevertheless, this difference could hardly alone account for females achieving nearly double the plasma OTA concentration of males. Immediately following the end of the 36 week OTA treatment period, decline in plasma OTA concentration in females followed a half-life of 9.7 days (Figure 3). 

### 3.2. Urinalysis

Measurement of urine output and of some standard chemical parameters in urine, that might reveal any marked effects on renal function of chronic OTA exposure, revealed some features of interest at the 22 and 36 week stages (Table 1).

**Table 1:** Mean urinalysis data from male and female hybrid rats at 5 and 8 month stages during maintenance on OTA-contaminated feed (5 ppm), and correspondingly from female controls. Creatinine values are mmol/L. Other solute parameters are expressed as µmol/mmol creatinine, particularly to accommodate the marked polyuria in OTA-treated females.

	Months on OTA or as controls	Rats (n)	Urine vol (mL)	Creatinine	Ca	Urate	Protein	Na	K	Phosphate	Urea
Male	5	4	7.98	12.04	0.24	0.16	0.20	11.87	23.67	0.24	90.55
(OTA)	8 A	4	11.37	9.18	0.30	0.12	0.19	18.31	24.09	0.89	91.45
	8 B	7	8.36	11.27	0.34	0.11	0.20	11.73	18.91	0.09	69.99
Female	5	3	14.67	3.69	1.38	0.23	0.06	17.80	36.54	2.24	119.9
(OTA)	8 A	3	20.67	3.74	1.30	0.23	0.08	26.40	35.08	1.97	123.2
	8 B	3	13.4	2.81	1.22	0.24	0.07	19.56	35.06	0.82	122.7
Female	5	3	9.20	6.63	1.12	0.15	0.04	12.72	24.96	2.27	93.80
(control)	8	3	10.83	5.08	1.38	0.16	0.04	16.87	24.89	1.52	105.6

Urine composition of control (untreated) females was of rather similar composition at 22 and 36 week stages, corresponding closely in some parameters to that for females given OTA, and providing a fairly stable basis for comparison. However, the OTA-treated females exhibited marked polyuria and a correspondingly lower creatinine concentration. Corrected protein output was doubled, and potassium concentration was consistently increased, suggesting other mild pathological outcomes.

**Figure 4:**
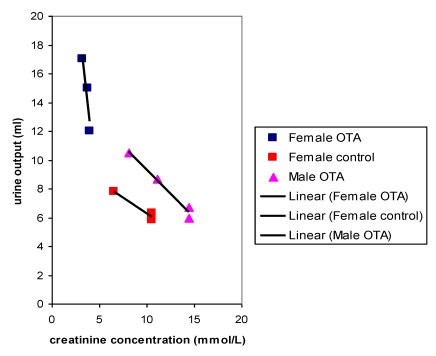
Comparative linear relationships between 18 hour urine output and creatinine concentration in male and female rats after 22 weeks with or without dietary OTA.

Plots of urine output versus creatinine concentration after 22 weeks treatment with OTA (Figure 4) showed a close correlation for OTA-treated males and for the female controls, as would be expected where OTA had no adverse effect. However, OTA-treated female data clustered separately to indicate marked polyuria. There was also a modest complementary disturbance in OTA-treated females particularly in potassium excretion (36.54 µmol/mmol creatinine, SD 1.28) compared with control females (24.96 µmol/mmol creatinine, SD 4.71). Potassium output in males was not significantly different from that in females. The disturbance in potassium excretion in OTA treated females would reasonably follow from polyuria caused by a mild effect of OTA more distally in nephrons.

**Figure 5:**
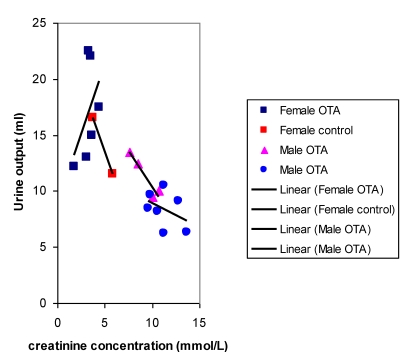
Comparative relationships between fitted curves for 18 hour urine output and creatinine concentration after 36 weeks of dietary OTA in male and female rats. Data from two measurements in males at an interval of one week overlap in a common inter-relationship. In contrast, data for females treated with OTA lack this correlation while it is present in that for untreated female controls.

After 8 months, near the end of OTA treatment, no marked polyuria was evident in females treated with OTA (Figure 5); correspondingly potassium excretion was not significantly disturbed.

In males, urine output (Table 1) did not differ from typical values and creatinine concentration was correspondingly in a normal range, according to unpublished data relating to experiments described in [3,4]. Protein was elevated typically by comparison with females, but both sodium and potassium values were close to those in female controls. Thus there was no consistent evidence of impaired renal function due to OTA treatment in these males. 

### 3.3. Pathology: males

**Figure 6:**
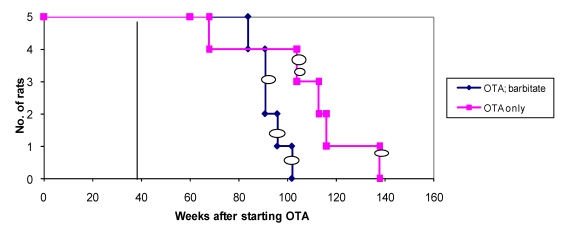
Natural lifetime survival of two groups of male rats, first given dietary OTA (5 ppm) for 36 weeks. After three weeks on normal diet, one group was also given sodium barbitate in drinking water for life. Oval symbols indicate stage of discovery of renal tumours.

Overall survival in the groups of male rats is summarised in Figure 6 and attributed graphically to incidence of renal tumours.

### 3.4. OTA without subsequent Barbitate

Apart from an inexplicable death (male at 68 weeks), no further deaths occurred until two years after the experiment started, and after all rats in the group given both OTA and barbitate (below) had deceased. One of the four remaining rats was euthanized because a subcutaneous sarcoma, which had gradually been developing during the previous 2 months, had become unacceptably large (29 g). Internally, bilateral renal tumours (still encapsulated to distort the kidneys by their dorsal or polar location) were found, but there was no obvious distant metastasis. DNA ploidy distribution in the sarcoma and the polar renal tumour was diploid, but the renal tumour histology showed carcinoma components. The dorsal adenoma was too small for ploidy analysis. 

Thereafter, the only case amongst all rats of typical mononuclear leukaemia became apparent, but only at 2 years and 4 months of age and with no other abnormality found at autopsy. 

In week 116, the 4th rat was euthanised because a chronic mouth absess was affecting feeding, but there were no renal neoplasms or other notable defects at necropsy. 

**Figure 7:**
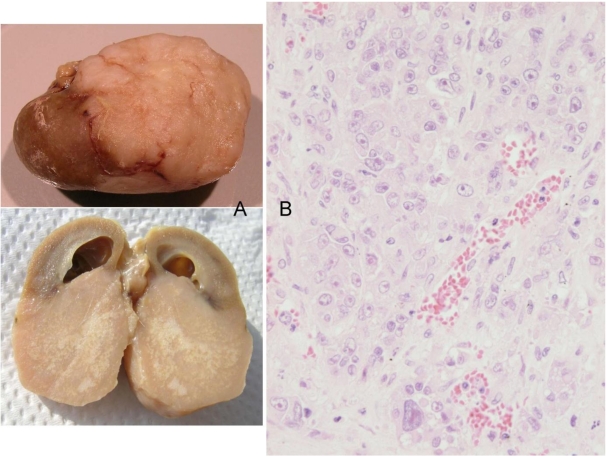
Male rat given the OTA regimen only. A, renal carcinoma causing organ distortion at the caudal pole; note renal papilla still entire. B, photomicrograph of H and E stained section of renal carcinoma; note disorganisation of tissue, frequent enlarged nuclei with prominent nucleoli and one very large nucleus.

Finally, the 5th animal was losing weight (Figure 2) at 2 years 10 months of age and was euthanised; the left kidney was cystic (typical of ageing rats) but the right kidney bore a large tumour that distorted the caudal pole (Figure 7A, total tumourous kidney weight 10 g). The tumour histopathology was typical of OTA-induced renal carcinoma (Figure 7B). Repeated attempts to measure DNA ploidy distribution failed for this tumour; nuclei were very large, with cleared centres after staining, and the scanning software could not recognise them. Thus 40% of these rats developed renal tumours and none became apparent until 2 years after the experiment commenced. 

### 3.5. OTA followed by Barbitate

In the male group given OTA and barbitate, the first loss was an animal euthanized at 84 weeks with a soft tissue tumour, the size and shape of a brazil nut, associated with muscle in the groin region. There were no other obvious abnormalities. 

Seven weeks later (1 year after barbitate treatment started), a second animal was found dead (inexplicably), and in a third moribund animal was revealed a large (30 g) right renal tumour, haemorrhage from which was the obvious cause of its moribund state (illustrated elsewhere, Figure 3 A,B in [16]. Extensive metastatic nodules were on abdominal serosal surfaces, but notably not on the bladder or in liver or lungs. DNA ploidy distribution in the renal carcinoma was aneuploid (nearly up to 8c) in tissue containing karyomegalic nuclei with prominent nucleoli. This is consistent in our experience with the findings of [17] which correlated aneuploidy with the histological characteristics of OTA-related renal carcinoma and its disorganized infiltrating cells. 

**Figure 8:**
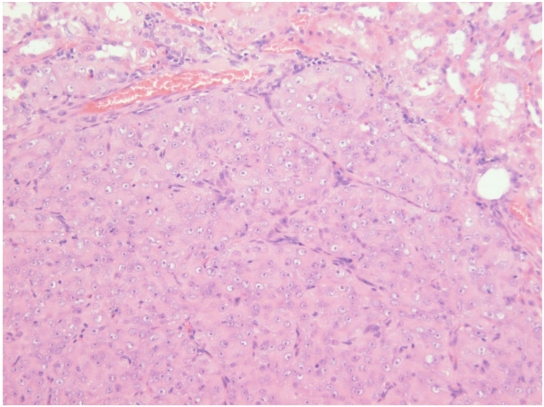
Photomicrograph of H & E stained section showing adenoma *in situ* in kidney. Note arrangement of cellular proliferation still within expanded renal tubules.

In week 96, ‘Multistix’ test on a few drops of urine from the remaining two rats revealed haematuria in one animal (weight 697 g), possibly indicating a renal tumour. The rat was euthanized; no gross renal abnormality was evident but longitudinal section of the right kidney revealed a tumour *in situ*, thereby explaining the haematuria. A quite early stage of renal tumourigenesis (adenoma histopathology, Figure 8) was thus observed, discovered only at about 2 years of age and after one year’s treatment with barbitate. The last rat, at 102 weeks after starting OTA exposure, had been losing weight (Figure 2) and was euthanised; a large left renal carcinoma (35 g) was evident. Metastatic nodules occurred on serosal surfaces in the abdomen and in the lungs. 

Thus 60% of rats had a unilateral renal tumour and 40% were metastasising carcinomas that became life-threatening just within 2 years of commencing the OTA exposure and one year after commencing the barbitate treatment.

### 3.6. Pathology: females

**Figure 9:**
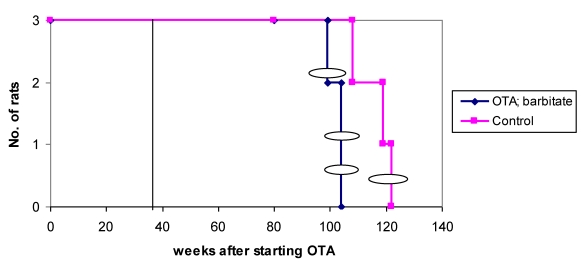
Natural lifetime survival of two groups of female rats. One was given dietary OTA (5 ppm) for 36 weeks. After three weeks on normal diet, the group was also given sodium barbitate in drinking water for life. The other control group was given normal diet for life. Oval symbols indicate time of euthanasia because of mammary tumour size.

Overall survival in the groups of female rats is summarised in Figure 9 and attributed graphically to incidence of renal tumours.

In the females given the combined OTA and barbitate regimen a single mammary tumour was first detected in each animal at week 87, nearly a year after barbitate treatment commenced. Progressive tumour proliferation required that these rats were euthanised 3–4 months later (Figure 2). Thus, at week 99 a thoracic region tumour was at risk of abrasion and ulceration; the tumour weighed 50 g.

**Figure 10:**
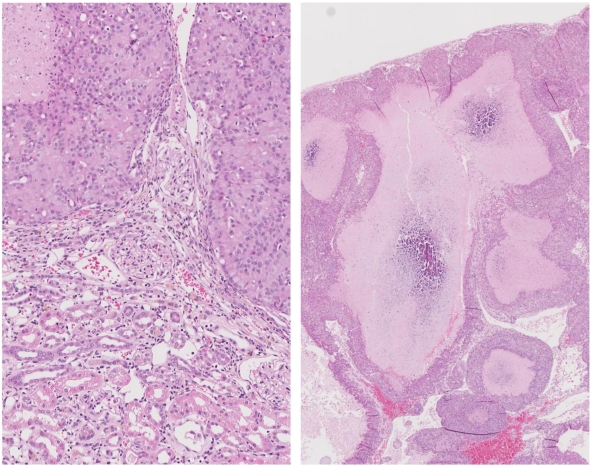
Photomicrographs of H and E section of female rat renal adenoma. Left, junction between tumour and kidney showing retained glomerular integrity close to tumour. Right, tumour containing large lacunae containing calcium-like deposits.

At week 104 the remaining two animals were euthanized because of their large mammary tumour. In one the tumour was in the left brachial region (~25 g, including white milk-like secretion associated with tissue surrounding a 2 cm diameter central hard white core); this rat also had a small renal tumour (10 × 12 mm), capsulated *in situ* but distorting the dorsal aspect of the kidney. Renal tumour histopathology was consistent with an adenoma enclosing two lacunae containing calcium-like deposits (Figure 10). The other rat had a huge, subcutaneous, encapsulated and highly vascular tumour in the right groin region (diameter 3.0–3.5 cm, weight 78 g). This mass also contained some milk but it was more cream-like in colour and consistency than that of the other animal. There was no other obvious pathological change.

Two of the 3 female controls were euthanised at weeks 108 and 119 but with no obvious pathological change. In the 3rd animal, a mammary tumour had been first detected in week 116, over 6 months later than those in the OTA-barbitate group, and grew to necessitate euthanasia 6 weeks later; the somewhat-flattened tumour was 3 cm in diameter and 1cm deep at the centre. There were no renal hyperplasias or other internal abnormality.

### 3.7. Comparative mammary tumour histopathology

**Figure 11:**
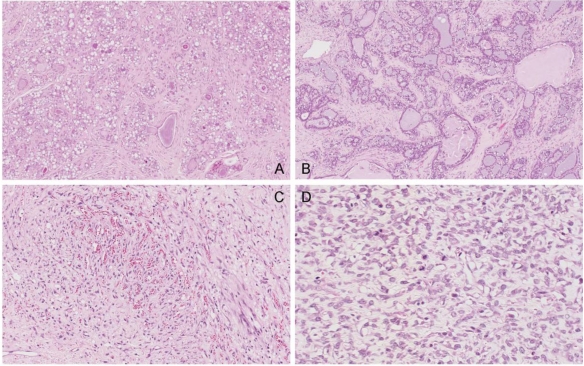
Photomicrographs of H & E section of female mammary tumours. A, fibroadenoma with secretory changes of the control; B, example of fibroadenoma of rats given the OTA-Barbitate regimen; C and D, angiosarcoma of a rat given the OTA-Barbitate regimen, illustrating regions rich in vascular proliferation (C) and with abundant mitoses (including abnormal forms) in atypical endothelial cells (D).

In the single tumour from a control rat, sections showed fibroadenoma with secretory change in the epithelial component, seen as cytoplasmic vacuolation and secretions in the acinar spaces (Figure 11A). There is also an unusual, somewhat vascular, nodular area of stromal cell proliferation. The cells in this fibrous area are not pleomorphic, although scattered mitoses are noted. The nodular component appears to be benign and this probably represents a prominent area of stromal hyperplasia in a fibroadenomatous background. DNA ploidy distribution in this tumour was diploid.

Similarly, amongst the mammary tumours of the treated group, the first to be studied at 99 weeks of age (87 weeks after OTA exposure commenced) was a fibroadenoma with a variety of morphological appearances, including some with tubular-type pattern and others with more extensive fibrous stroma. In particular there are extensive (7 mm diameter) areas of secretory change with intracytoplasmic vacuoles and with secretion into the lumina (Figure 11B). Dilated spaces with surrounding macrophages are noted, in keeping with leakage of secretory contents and inflammatory reaction. DNA ploidy distribution in this tumour was diploid. The second to be studied, at 104 weeks after OTA exposure commenced, is also a fibroadenoma, with extensive secretory changes and focal fibrosis.

However, the third tumour was a large, extensively infracted spindle-cell tumour with irregularly-shaped, thin-walled vascular channels admixed with small capillaries (Figure 11C, D). The blood vessels are focally anastamosing. The endothelial cells are plump in areas, although flat and relatively unremarkable in others. There is no true necrosis but scattered mitoses, including abnormal forms, are seen and this is interpreted as a malignant vascular tumour (angiosarcoma). DNA ploidy distribution in this tumour was also diploid. 

## 4. Discussion

Whereas barbitate treatment only slightly accelerated the development of OTA-induced renal tumours, all tumours in the barbitate group had been found before any without barbitate. Further, the bilateral renal tumours found in an animal euthanized because of a subcutaneous sarcoma were seen earlier than they would have been when they became the primary cause of morbidity. This potentially differentiates a modest promotional effect of barbitate, but that interpretation lacks statistical power. Further, there was still a 12-month latency period between the end of OTA exposure and the discovery of the first renal tumour in an animal treated continuously with this tumour promoter. In any case, any slight effect of barbitate on the rate of expression of the highly specific renal tumourigenesis initiated by OTA in males was less marked than its apparent acceleration of mammary tumourigenesis in females. Diagnosis of an angiosarcoma of vascular origin in a mammary tumour of a rat exposed to the OTA-barbitate regimen is apparently a novel finding for this animal.

The nearly two-fold difference in measured plasma OTA concentration between genders, with close similarity between replicates, is greater than can be explained by the slightly higher OTA intake for females in the present study. This expression of marked gender difference following chronic exposure to OTA is even matched in single dose pharmacokinetics in Fischer rats [18]. The concept of an exclusively-male uptake system for proximal tubular epithelia by binding of OTA to an α2u-globulin [16], thereby augmenting the uptake mechanism(s) common to male and female rats, could account for the lower plasma OTA concentration achieved in the male. Additional to the OTA uptake *via* the carrier protein there is also the direct excretion of some α2u-globulin-OTA complex in urine, where it may be recognised by gel electrophoresis. Further, the greater body-mass accumulation in OTA-treated males (already evident at the 22 week stage, Figure 2) may not yet have been associated with any significant deviation from the constant ratio of blood plasma volume to body weight in males and females measured for rats up to 400 g [19]. However, subsequently, as mature males gradually deposited abdominal fat, their ratio may have changed. Nevertheless, the higher circulating concentration of OTA in female blood might allow greater influence on mammary tissue that is naturally a site of potential tumourigenesis with age. Notably by the start of barbitate treatment, plasma OTA concentration had already followed two cycles of an approximately 10-day half-life decline, so that significant nephrotoxin would have long since been cleared from circulation and tissues through most of the subsequent barbitate treatment. 

Sensitivity of females, expressed covertly as polyuria after 22 weeks of OTA treatment, was apparently resolved by the time that treatment regimen ceased. Notably, marked polyuria was also recorded after 7 and 21 days of OTA treatment given by oral gavage to male F344 rats [20], implying that this response is a consistent indicator of mild temporary renal damage in young rats in response to OTA. That a change in potassium excretion was concomitant with polyuria is also consistent with OTA targeting distal nephron regions associated with water and cation homeostasis. Specific histopathological change in renal papilla in high OTA dose female rats in the NTP study [16] could further corroborate a distal nephron target in females.

In males, in response to their exposure to 5 ppm dietary OTA, the overall incidence of renal tumours (50% in animals and 30% in kidneys), and 30% as renal carcinomas, is generally consistent with published dose/response data. In the present pilot study it is not possible to conclude any significant effect of subsequent exposure to barbitate on renal tumour incidence, particularly if the bilateral occurrence without barbitate is considered (30% of kidneys with tumours in each group). Nor is it possible to perceive any notable promotion influence of barbitate on the time course for renal tumourigenesis that is already fully programmed by the 36 weeks of exposure to dietary OTA.

The findings concerning mammary tumours require more detailed discussion with respect to the only two previous lifetime studies on OTA and mammary tumours. The study of [21], the protocol of which was closely linked with a previous publication [2], subsequently explained [22], was based on OTA administration (400 µg/kg in 0.1 M sodium bicarbonate) by intragastric intubation 3 times per week for life (up to two and a half years). Although administered somewhat differently from the present experiment, predictably delivering relatively more free OTA to tissues during the periods of intermittent acute surges of circulating toxin, the mean daily OTA intake (e.g. 57 µg in a 333 g rat) was almost the same as that for females in the present experiment, and only slightly more than in the NTP high dose group [1]. The contrasting responses of Dark Agouti (DA) and Lewis females in producing mammary tumours, described as strain-specific, may follow from markedly shorter plasma OTA half-life (about 3 days) measured in males of the DA strain [4]. In contrast, the albino F344 strain males had a plasma OTA half-life of about 10 days [4], as did the hybrid females in the present study. Such differences could be reflected in the steady state plasma OTA concentration predicted for the same dosing regimen, whereby DA females would achieve a lower plasma concentration than Lewis females. This might then impose a lower toxicological pressure of the correspondingly smaller traces of free OTA in the blood circulating through DA mammary tissue.

It has been suggested that different gender expression of cytochrome P_450 _enzymes could partly account for different renal tumourigenic sensitivity *via* differential metabolic transformation of OTA prior to formation of DNA adducts [23]. However it is difficult to use findings in aged (2 years) survivors to explain the marked differential in genotoxic initiation of renal carcinogenesis, necessarily put in place at least a year earlier, in rats while expressing their full sexuality. It is difficult also to relate these metabolic findings to the gender difference in plasma OTA concentration after the chronic OTA exposure here, or to that described for the acute pharmacokinetics after a single OTA dose [18]. 

The title of the Son *et al*. study [21] with DA and Lewis rats implies focus on mammary tumours. However, that topic forms only a minor part of the report, but the publication does give growth curves and survival data that are complementary to the study of [2]. Nevertheless, in scoring the incidence of mammary proliferations there is no indication of their magnitude (microscopic or macroscopic), nor of the stages in later life at which they were discovered. Thus their significance to the animals’ longevity is unclear. However, in the present findings with a small number of untreated female albino rats, occurrence of a single tumour in the oldest animal conformed to the frequency of such neoplasms in ageing rats [1]. In contrast, the OTA-barbitate treatment regimen seemed to produce a synchronous earlier appearance of tumours in all females. The classic NTP study on OTA reports that “Incidence of fibroadenomas in high dose female rats was significantly greater than that in vehicle controls; histologically they were typical mammary gland fibroadenomas with a mixture of glandular and stromal elements”. Animals with multiple mammary tumours were more frequent in high OTA dose females. However, no details of tumour magnitide are given. 

In the present pilot study, diagnosis of the control mammary tumour and two of the treated group tumours as fibroadenoma is unremarkable. Further, that all treated females developed a mammary tumour after OTA at a relatively high dosage would not be inconsistent with findings both in the NTP study in F344 rats [1] and in the subsequent study with Lewis rats [2]. However, the predominant fibroadenoma histopathology serves as a norm against which to contrast the unusual malignant angiosarcoma in the group given the OTA-barbitate regimen. Generally in ageing female rats spontaneous mammary tumours are benign fibroadenomas; less than 10% are adenocarcinomas [24,25,26]. 

Differentiation of the OTA and barbitate influences in the aetiology of the angiosarcoma is difficult but, assuming that barbitate is only a tumour promoter [8], it would follow that the 36 weeks exposure to OTA, as opposed to the ‘lifetimes’ in the previous studies, was also sufficient to initiate this female mammary neoplasm, but that promotion occurred only after a long latency, as in the renal tumours of their male siblings. Barbitate may therefore have had an effect in advancing rat mammary tumour development to a significant degree (by about 6 months in a potential maximum lifetime of about 2.5 years) and in influencing occurrence of the unusual angiosarcoma. However, a role for OTA in initiating this tumour can not be excluded. Nevertheless, the fact that the mammary tumours remain diploid within the detection limit of the ploidy analyser (approximately 1% cellular DNA content) suggests that barbitate does not cause gross chromosomal instability.

Unfortunately in the publication of [21], although incidence of ‘mammary lobular hyperplasia (6/19) and atypical mammary hyperplasia (4/19)’ was recorded, there is no illustration of these neoplasms. It is assumed that they comply with guidelines of STP/ARP/AFIP for toxicologic pathology [27]. The only incidence of mammary proliferative lesions elevated above that in controls and attributable to the continuous OTA treatment was in female Lewis rats. In citing the findings of [21], a subsequent publication [28] recommended further study of potential toxicity of OTA to mammary tissue and applicability to human populations. The present pilot study may not further this objective because of the difficulty in differentiating the OTA and barbitate components of the females’ treatment. However, considering the measured plasma OTA half-life, circulating OTA would have already decreased by a factor of 10 during the month after ceasing the contaminated feed. Clear evidence of long latency of an OTA effect on mammary tumourigenesis would have to be shown for the present OTA exposure to be a plausible cause of the tumours diagnosed nearly a year later. The present synchronous advance of mammary tumour expression is more likely to have been influenced by the barbitate, which also seemed to cause the body weight gain (Figure 2).

In summary, male kidneys in which carcinogenesis had been initiated silently by OTA treatment apparently responded to the barbitate tumour promoter by slight acceleration in tumour proliferation, but not to the extent shown by female mammary tumours. However, the apparent effect in males can not compare with the extent that barbitate alone can rapidly promote renal tumours either spontaneously in Eker rats [9] or in rats after exposure to only a single dose of nickel [8]. Development of male rat renal tumours initiated by OTA probably *via* its genotoxicity [29] and with an above-threshold exposure [30], therefore seems rather strongly linked to ageing and its natural decline in tumour suppression. However, although it is difficult in the present pilot study to differentiate putative influences of OTA and barbitate in initiating mammary tumours, the observed effect may have been caused by barbitate exacerbating the natural tendency of ageing females to produce mammary tumours. Even so, the tumours were only diagnosed after about 10 months of barbitate treatment. There seem therefore no adverse implications for risk in human female health from trace exposure to OTA since application of the literature on OTA-associated rat mammary tumours (designated as fibroadenomas) to human risk assessment is presently limited by the marked absence of any experimental dose/response correlation; only the highest dose was effective in the NTP study [1]. Nevertheless, the present findings should encourage lifetime studies in female rats, focused on mammary tumourigenesis in animals given barbitate tumour promoter after an OTA regimen sufficient for renal tumourigenesis in males. For males the worst effect of silent chronic OTA exposure might only be for renal tumourigenesis during ageing. However, caution in extrapolating from male rat renal toxicological data *in vivo* to humans should be taken to allow for male rat characteristics affecting OTA pharmacokinetics that might just be exclusive to this laboratory animal [16]. 
